# Magnetic resonance imaging features of hippocampus and mechanism of neurocognitive dysfunction for antiepileptic drugs in treatment of depression rats

**DOI:** 10.1080/21655979.2021.2018537

**Published:** 2022-02-11

**Authors:** Tuxiu Xie, Ran Li, Xiaobing Long, Jun Chen, Lu Ye, Jing Wang, Guijun Jiang, Jingjun Lv

**Affiliations:** a Department of General Practice, Renmin Hospital of Wuhan University, Wuhan, Hubei Province, China; b School of Basic Medical Sciences, North China University of Science and Technology, Tangshan, Hebei Province, China; c Department of Emergency, the Center of Emergency and Critical Care Medicine, Renmin Hospital of Wuhan University, Wuhan, Hubei Province, China; d Department of Radiology, Renmin Hospital of Wuhan University, Wuhan, Hubei Province, China

**Keywords:** Sodium valproate, magnetic resonance imaging images, neurometabolites, JAK1/STAT3 pathway, inflammatory factors, cognitive function

## Abstract

To explore the effects of antiepileptic drug sodium valproate on magnetic resonance imaging (MRI) images, neurological cognition, and JAK1/STAT3 pathway in hippocampus of rats with depression, 30 Sprague Dawley (SD) rats were included. The depression model (DM) was prepared through the chronic stress restraint test. Some model rats were injected with 10 mg/kg sodium valproate into abdominal cavity before modeling (RT group)), and healthy rats were selected as controls (healthy control (HC) group). Depth of split brain was greatly increased in DM group, and nitrogen-acetyl aspartic acid (NAA)/creatine (Cr), glutamic acid (Glu)/Cr, and choline (Cho)/Cr ratios were greatly reduced (*P* < 0.05). Behavioral test results showed that sugar water preference rate, escape latency, and divergence index in DM group were greatly reduced (*P* < 0.05), and cumulative immobility time, target quadrant stay time, and number of crossings in forced swimming and tail suspension were prolonged dramatically (*P* < 0.05), with no difference between the two groups (*P* > 0.05). Expression levels of interleukin 1β (IL-1β) and interleukin 6 (IL-6) in hippocampus of DM group were obviously increased (*P* < 0.05), and expression levels of JAK1 and STAT3 were decreased visibly (*P* < 0.05), with no difference between the two (*P* > 0.05). In summary, anti-epileptic drug sodium valproate effectively improves hippocampal volume characteristics and memory and neurocognitive dysfunction of depression models.

## 1. Introduction

Depression is also called depressive disorder. There are many factors that cause depression, and patients mainly show mood disorders. Therefore, depression has become a very common major mental illness [1]. The pathogenesis of depression is very complicated and may be related to factors such as genetics, emotions, and social environment. In recent years, more and more studies have confirmed that chronic stress can also cause depression, and chronic stress can cause changes in brain structure and function such as the hippocampus, prefrontal cortex, and amygdala [2,3]. Brain imaging can provide further insights into the pathogenesis of depression from the perspectives of brain structure and neuronal material metabolism. Using magnetic resonance imaging (MRI) for depression research can find characteristic changes in the brain structure or brain function of patients [4]. Magnetic resonance spectroscopy (MRS) can perform metabolic analysis of neurobiochemical substances in patients with depression [5].

The hippocampus is a very important part of the limbic system, located between the thalamus and the medial temporal lobe, and it participates in cognitive function processes such as emotion and learning. The abnormal function of the hippocampus is related to depression, anxiety, and other affective disorders, and the hippocampus is also the main area of stress injury [6]. Moreover, chronic stress can cause apoptosis and necrosis of glial cells in the hippocampus. Studies have confirmed that activation of the Kv7 pathway can effectively improve the mental symptoms such as anxiety and mania, and can better the learning and memory disorders in animal models of epilepsy or Alzheimer’s [7]. The Kv7 channel is an important branch of the potassium channel superfamily, and is a unique ligand-regulated voltage-dependent potassium channel. It is encoded by the KCNQ gene, and is coupled to the M choline receptor at the same time. Activation of the M receptor can turn it off, so it is also called the M channel. Sodium valproate is the drug of choice for major primary seizures and minor absence seizures. In addition to anti-epilepsy, it can also be used to treat febrile seizures, dyskinesias, chorea, and other diseases. Sodium valproate drugs can activate the Kv7 pathway to improve hippocampal-dependent spatial learning and memory impairment caused by stress [8]. At present, sodium valproate has been approved by the Food and Drug Administration (FDA) of America as a new type of anti-epileptic and anti-convulsant clinical drug. However, it is still unclear whether sodium valproate can be used in the treatment of depression caused by chronic stress.

Based on this, a model of rats with depression through chronic stress was established and gave sodium valproate injection therapy. First, the changes in depression-like behavior and cognitive function disorders in rats were explored. Then, MRI images and MRS scans were used to analyze the changes in the structure of the rat hippocampus and the metabolic changes of neurobiochemical substances. Finally, the changes in inflammatory factors and JAK1/STAT3 pathways in the hippocampus of rats were detected. The objective of this study was to understand the effect of anti-epileptic drug sodium valproate in the treatment of chronic stress-induced depression-like behavior and cognitive dysfunction and its regulatory mechanism. It aimed to provide new ideas for the treatment and prevention of depression and the development of antidepressant drugs, and give reliable basic experimental data for the clinic.

## 2. Materials and methods

### 2.1 Experimental materials

The clean-grade healthy Sprague Dawley (SD) rats were purchased from Shanghai Legian Biotechnology Co., Ltd.; sodium valproate was purchased from Shanghai Rechemscience Co., Ltd.; 10% chloral hydrate was purchased from LANSO (Shanghai); Sucrose, radio-Immunoprecipitation Assay (RIPA) lysis solution, bicinchoninic acid (BCA) protein quantitative detection kit, tris-buffered saline tween (TBST) buffer, and efficient chemiluminescence (ECL) chemiluminescence detection kit were purchased from Thermo Fisher Scientific; 5% bovine serum albumin (BSA) was purchased from Solarbio Life Sciences (Beijing); and the protein primary and secondary antibodies were purchased from Abcam in UK.

### 2.2 Preparation of an animal model of depression

In the experiment, 30 rats were randomly raised in cages, and ensured with sufficient drinking water and food. The temperature of the animal laboratory environment was controlled at 23 ± 1°C, the relative humidity was controlled at 50 ± 3%, and it was kept quiet and ventilated for 12 hours with alternative day and night. After adapting to feeding for 7 days, the rats were randomly divided into a healthy control (HC) group, n = 10), a depression model (DM) group, n = 10) and a sodium valproate treatment group (RT group, n = 10). The rats in the DM group and the RT group were restrained in plastic tubes from 10:00 to 14:00 every day. They were kept ventilated to dissipate heat and prohibited from eating or drinking. The rats were restrained for 21 days and weighed every 1 day. Rats in RT group were injected intraperitoneally with 10 mg/kg sodium valproate after the completion of the model, while rats in HC and DM groups were injected with the same amount of normal saline intraperitoneally for 14 days. The rat depression model was prepared by referring to the reference [9].

All experimental operations were performed in accordance with the relevant regulations of the Animal Ethics Committee of the Renmin Hospital of Wuhan University.

### 2.3 MRI scanning and analysis

After the rats were fully anesthetized by intraperitoneal injection of 10% chloral hydrate, a 3.0 T MRI scanner was used to scan the brain regions of the rats. The MRI parameters were set to T2 weighted imaging (T2WI); the time of repetition (TR) was 3000 ms; the time of echo (TE) was 40 ms, 80 ms, and 120 ms; and the domain was 3.5 cm. A computer was used to automatically calculate the hippocampus volume on both sides. The calculation equation was: $HC_{1}\times T+HC_{2}\times T+HC_{3}\times T+\cdots +HC_{n}\times T$: where HC was the area of the hippocampus in the axial position, and T referred to the layer thickness and spacing [10].

### 2.4 MRS sequence scanning and analysis

About 2 mm × 2 mm × 2 mm of rat bilateral hippocampus and prefrontal cortex were taken as the region of interest (ROI). After advanced shimming, the PRESS sequence was selected for 1 H-MRS scanning, and the parameters were set. The TR was 2000 ms; the TE was 35 ms; the sampling point was 1024; and the sampling time was 35 minutes. The Topsin 5.0 software was selected for neurometabolites data processing and image reconstruction. The nitrogen-acetyl aspartic acid (NAA) 2.0 ppm, choline (Cho) 3.22 ppm, glutamic acid (Glu) 2.35 ppm, inositol (MI) 3.56 ppm, and creatine (Cr) 3.0 ppm were calculated.

### 2.5 Depression-like behavior test

The sugar water preference was performed as follows. The drinking water ingested by rats was replaced daily with 1% sucrose solution, and the rats were fed adaptively for 24 hours. Subsequently, they were reared in a single cage and randomly placed water bottles with the same appearance in the cages, each containing the same quality of drinking water and 1% sucrose solution. Next, the horizontal position of the water bottles was changed once during the experiment, Then, the reference value of each rat’s preference for sugar water within 3 days was recorded. After that, it officially entered the sugar water preference testing stage, and recorded the percentage of preference for sugar water within 24 hours. No fasting was allowed in the testing stage. The sugar water preference rate can be calculated with $V_{sw}/\left(V_{sw}+V_{w}\right)$, of which *V_sw_* was the amount of sugar water consumed, and *V_w_* referred to the amount of drinking water consumed [11].

The forced swimming test was performed as follows. The test rats were placed in a transparent plexiglass hollow cylinder with a height of 60 cm, a diameter of 20 cm, and a water depth of 30 cm. The water level was 30 cm above the cylinder, and the water temperature was maintained at 25 ± 2°C. After the start of the test, the rats were gently placed in the water and allowed to swim freely for 2 minutes. The cumulative immobility time of the rats was recorded in the following 5 minutes. After each rat completed the test, the water in the device had to be replaced [12].

The tail suspension test was performed as follows. The tape was adopted to fix the rat’s tail about 1 cm to the cross arm of a rectangular iron frame 60 cm high and 70 cm wide, and make its head hang downward. In addition, it should keep the rat’s nose about 20 cm away from the ground, and ensure that the experimental environment was quiet. The cumulative different time that the rat stopped struggling within 5 minutes after 6 minutes was calculated [13].

### 2.6 Morris water maze test

The main body of the Morris water maze was a circular water tank with a height of 45 cm and a diameter of 90 cm. Tap water was injected and a platform was placed on the level of the first quadrant. The water temperature was maintained at about 24°C. The rats were placed into the water from different quadrants to train for four consecutive days, with a training interval of 20 minutes and 4 times a day. If the rat failed to find the hidden platform after 1 minute, it was guided to stay on the platform for 30s, and the escape latency was recorded as 60s. After 4 days of training was completed, the original platform was removed, the rat was placed into the water from the III quadrant to allow to swim freely for 1 minute. The target quadrant stay time and number of crossings were recorded [14].

### 2.7 Novel object recognition (NOR)

The test device was made of a gray square wooden box. A 40 W light bulb and a camera were placed 120 cm above the device to record the activities of the rats in the wooden box. Firstly, the rats were placed in a wooden box for 5 minutes to move freely. After 24 hours, the cylinder (with the height of 6 cm and radius of 2 cm) and square glasses (with the length of 4 cm and the height of 6 cm) were placed on the two corners of the wooden box, about 5 cm away from the corner of the box. Subsequently, the rat was placed in a wooden box with the corner of the object that had never been placed with its back facing away. When the tip of the rat’s nose touched the object or the distance was ≤ 2 cm for 20s, it was deemed that the object was successfully explored. After 1 hour, the square glass cup was replaced with a sphere (about 5 cm in diameter), and the times when the rat touched the old and new objects within 5 minutes were recorded. After each test, it was necessary to clean up the feces in the wooden box and wipe all objects with alcohol. The divergence index of the rat was calculated according to the equation: $\left(t_{N}-t_{F}\right)/(t_{N}+t_{F}$, where *t_N_* was the time of contacting new things, and *t_F_* referred to the time of contacting old things [15].

### 2.8 Western blot

After the rats were decapitated, their brain tissues were taken and weighed, the hippocampus was separated, and RIPA lysate was added at a ratio of 1:9 (mg/μL) to extract the total protein in the tissues. The BCA kit was adopted to determine the protein concentration, and the protein concentration was adjusted to a uniform. After a separation gel suitable for the molecular weight of the target protein was prepared, 50 μg of the protein was collected and loaded, and then performed with electrophoresis at 80 V constant pressure at room temperature until the target band was separated. After the electrophoresis, the target protein was transferred to the polyvinylidene fluoride (PVDF) membrane, and then placed in ice water and maintained a constant current of 280 mA for electrophoresis. After transfer, it was placed in the Ponceau stain for 1 minute, and the target protein band was cut out. Then, the Tris Buffered Saline Tween (TBST) rinsing and dyeing solution was adopted. 5% BSA was placed into the solution, which was sealed in a shaker at room temperature for 2 hours. The diluted primary antibodies IL-1β (1:1000), IL-6 (1:1000), JAK1 (1:1000), STAT3(1:1000), and β-actin (1:1000) were added and then incubated overnight in a refrigerator at 4°C. After the membrane was rinsed with TBST, it was added with horseradish peroxidase-labeled goat anti-mouse or goat anti-rabbit secondary antibody (1:5000), and incubated at room temperature for 2 hours. After the membrane was rinsed with TBST, it should add some electro-chemiluminescence (ECL) developer solution, and the gray value of the target protein band was analyzed using ImageJ software [16].

### 2.9 Statistical analysis

The data obtained in all experiments were expressed by mean ± standard deviation (SD), and the difference among different groups was compared by one-way analysis of variance in SPSS19.0. It was considered that *P* < 0.05 meant a statistically significant difference, and *P* < 0.01 suggested an extremely and statistically significant difference.

## 3. Results

### 3.1 The effect of sodium valproate on weight growth in rats with depression

**Figure 1.**
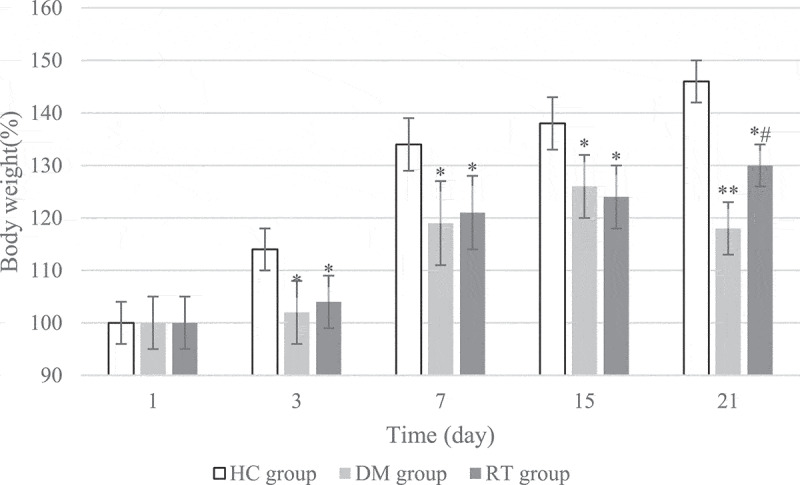
The effect of sodium valproate on weight growth in rats with depression. * and ** suggested *P* < 0.05 and *P* < 0.01 in contrast to the HC group, respectively; and # indicated *P* < 0.05 compared with the DM group.

The changes in the weight growth rate of rats in each group during the 21 days of modeling were detected, and the results are shown in Figure 1. On the 1^st^ day of model establishment, there was no great difference in the weight growth rate of rats in the HC group, DM group, and RT group (*P* > 0.05). On the 3^rd^, 7^th^, and 15^th^ day of modeling, the weight growth rates of rats in the DM group and the RT group were dramatically reduced compared with the HC group (*P* < 0.05), but there was no obvious difference in the weight growth rate of the rats in the DM group and the RT group (*P* > 0.05). On the 21^st^ day of modeling, the weight growth rate of rats in the DM group was remarkably reduced compared with the HC group (*P* < 0.01), and the weight growth rate of rats in the RT group was greatly reduced (*P* < 0.05); and the weight growth rate of rats in the RT group increased notably compared with the DM group (*P* < 0.05).

### 3.2 The effect of Sodium valproate on the hippocampal volume of rats with depression

**Figure 2.**
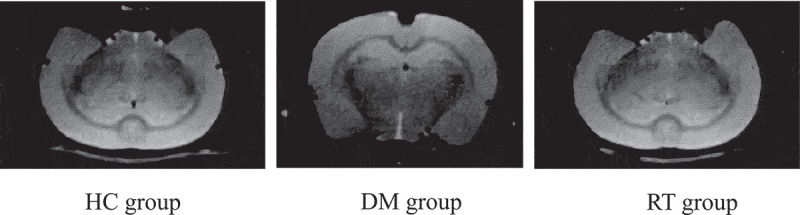
Brain MRI images of rats in each group.

The effects of sodium valproate on hippocampal volume of rats with depression were analyzed by MRI images, and the results are given in Figure 2. The results revealed that the bilateral hippocampus of the rats in the HC group, the DM group, and the RT group were basically symmetrical, but the right hippocampus of rats in the DM group was slightly smaller than the left one. In addition, the split brain depth of rats in the DM group increased obviously, which was greater than that of the HC group and RT group.

### 3.3 Effect of sodium valproate on neurometabolites in hippocampus of rats with depression

**Figure 3.**
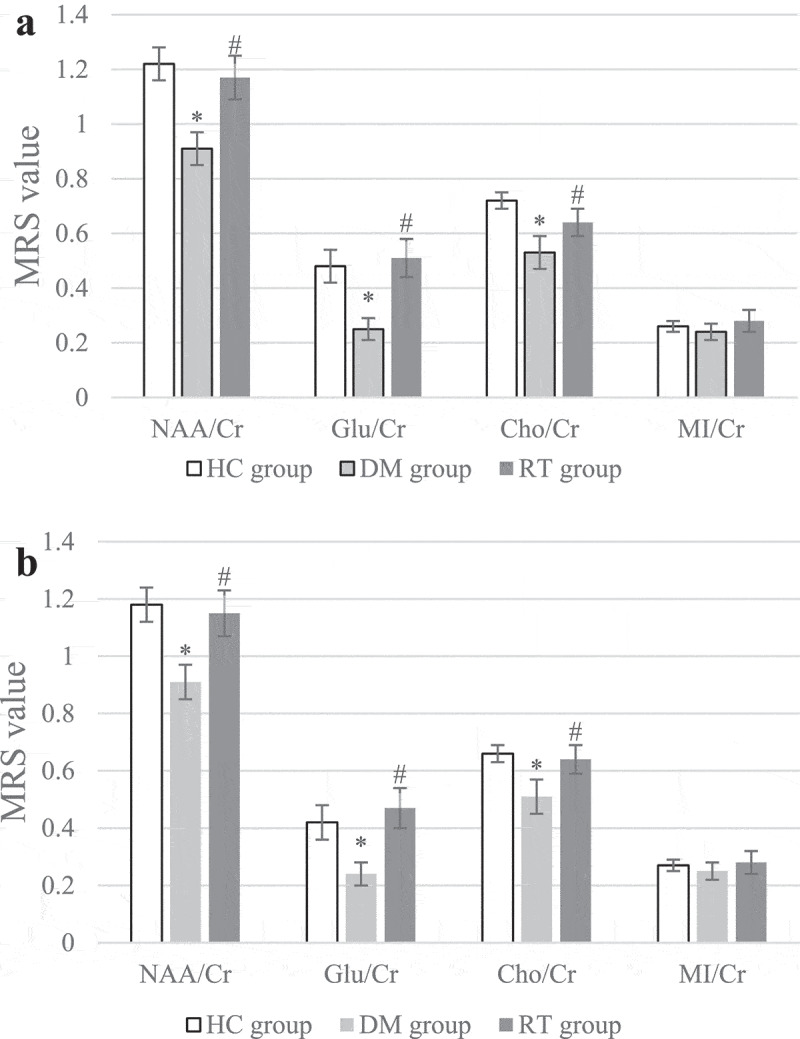
Comparison on differences in MRS values of bilateral hippocampus of rats in each group.

Note: Figure A shows the statistics of the neurometabolites MRS values in the left hippocampus; and Figure B is the statistics of the neurometabolites MRS values in the right hippocampus. * suggested *P* < 0.05 compared to the HC group, and # indicated *P* < 0.05 compared to the DM group.

MRS was used to scan the differences in neurometabolites levels in the bilateral hippocampus of rats, and the results are shown in Figure 3. Figure 3(a,b) illustrated that compared with the HC group, the NAA/Cr, Glu/Cr, and Cho/Cr values of the left and right hippocampus of the DM group were greatly reduced (*P* < 0.05); compared with the DM group, the NAA/Cr, Glu/Cr, and Cho/Cr values of the left and right hippocampus of the RT group increased remarkably (*P* < 0.05); while the HC group and the RT group showed no obvious difference in bilateral hippocampal NAA/Cr, Glu/Cr, and Cho/Cr values (*P* > 0.05). There was no visible difference in MI/Cr values in the bilateral hippocampus of rats in each group (*P* > 0.05)

### 3.4 The effect of sodium valproate on depression-like behavior in rats with depression

**Figure 4.**
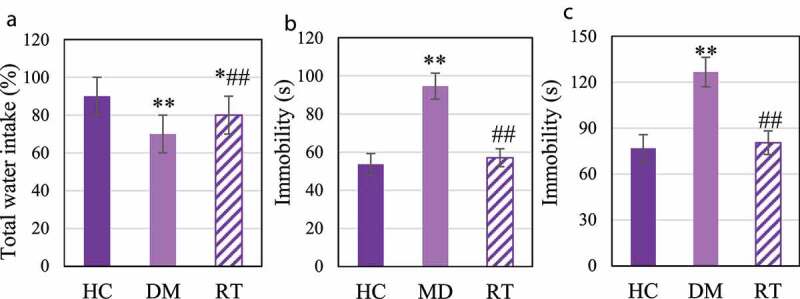
The effect of Sodium valproate on depression-like behavior in rats with depression.

The sugar water preference test, forced swimming test, and tail suspension test were performed to explore the effects of Sodium valproate on depression-like behavior in rats with depression. The results are shown in Figure 4. Figure 4(a) illustrates that compared with the HC group, the sugar water preference rate in the DM group was extremely reduced (*P* < 0.01), and that in the RT group was much lower (*P* < 0.05); and compared to the DM group, that in the RT group was notably increased (*P* < 0.05). Figure 4(b,c) disclosed that compared to the HC group, the cumulative immobility time of the DM group rats in the forced swimming and tail suspension tests increased dramatically (*P* < 0.01); that in the RT group was decreased greatly in contrast to the DM group (*P* < 0.05); while there was no remarkable difference in the cumulative immobility time for rats between the HC group and the RT group (*P* > 0.05).

Figure A was the statistics of sugar water preference rate; Figure B was the statistics of the cumulative immobility time in the forced swimming test; and Figure C showed the statistics of the cumulative immobility time in the tail suspension test; * and ** suggested *P* < 0.05 and *P* < 0.01 compared with the HC group, respectively; and ## meant *P* < 0.01 compared with the DM group.

### 3.5 The effect of Sodium valproate on rats with depression memory function and cognitive function

**Figure 5.**
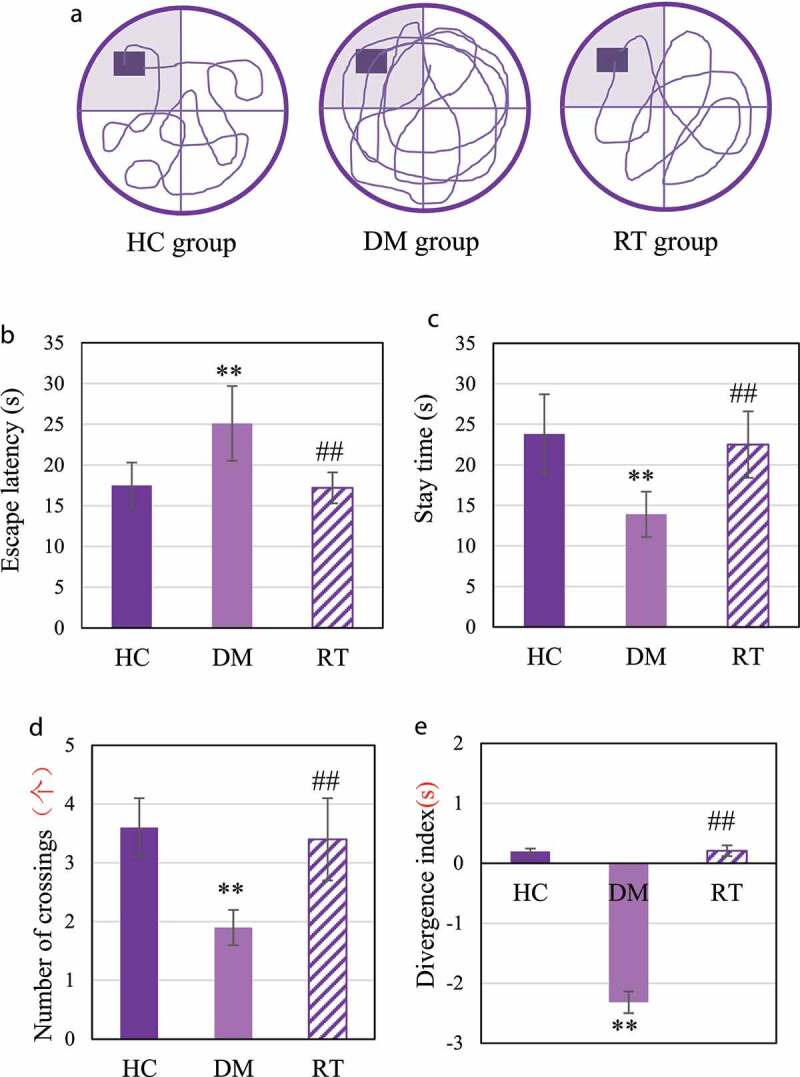
The effect of sodium valproate on rats with depression memory function and cognitive function.

Note: Figure A was the swimming route of the rat in the Morris water maze; Figures B, C, D, and E showed the statistical results of escape latency, target quadrant stay time, and number of crossing, and the divergence index, respectively. ** and ## suggested *P* < 0.01 in contrast to HC and DM groups, respectively.

The Morris water maze test was performed to analyze the effect of sodium valproate on memory function of rats with depression, and the results were shown in Figure 5. Figure 5(a) discloses that the driving path of the DM group in the maze was longer than that of the HC group and the RT group. Figure 5(b) reveals that the escape latency of rats in the DM group was extremely longer than that in the HC group (*P* < 0.01) and the RT group (*P* < 0.05); and there was no visible difference in escape latency between the HC group and the RT group (*P* > 0.05). Figure 5(c,d) suggested that the quadrant stay time and number of crossings in the DM group were much less in contrast to those in the HC group (*P* < 0.01) and the RT group (*P* < 0.05). In addition, there was no obvious difference between the HC group and the RT group in the quadrant stay time and number of crossings (*P* > 0.05).

NOR was to detect the effect of sodium valproate on rats with depression cognitive function, and the results are shown in Figure 5e. The divergence index of rats in the DM group was extremely lower compared with the HC group (*P* < 0.01) and the RT group (*P* < 0.05); and the divergence of the rats in the HC group and the RT group was not greatly different (*P* > 0.05).

### 3.6 The effect of sodium valproate on the expression of inflammatory factors in the hippocampus of rats with depression

**Figure 6.**
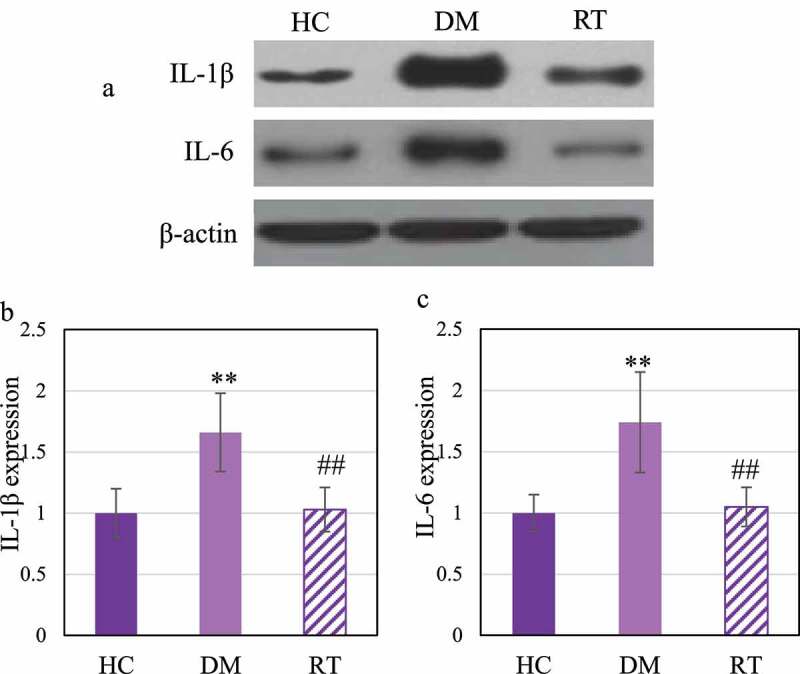
Comparison on differences in the expression levels of IL-1β and IL-6 protein in the hippocampus of rats in each group.

Note: Figure A shows the Western blot strip; Figure B and Figure C show the levels of IL-1β and IL-6, respectively. ** and ## suggested *P* < 0.01 in contrast to HC and DM groups, respectively.

Western blot was adopted to detect the effect of Sodium valproate on the protein expressions of interleukin (IL) −1β and IL-6 in hippocampal inflammatory factors of rats with depression. The results are illustrated in Figure 6. Figure 6(a) reveals that IL-1β and IL-6 levels in the DM group increased greatly. Figure 6(b,c) illustrated that the IL-1β and IL-6 protein expression levels in the DM group increased observably compared with the HC group (*P* < 0.01); those in the RT group greatly decreased in contrast to the DM group (*P* < 0.05), and those in the HC group and the RT group were not remarkably different (*P* > 0.05).

### 3.7 Effect of sodium valproate on JAK1/STAT3 pathway in hippocampus of rats with depression

**Figure 7.**
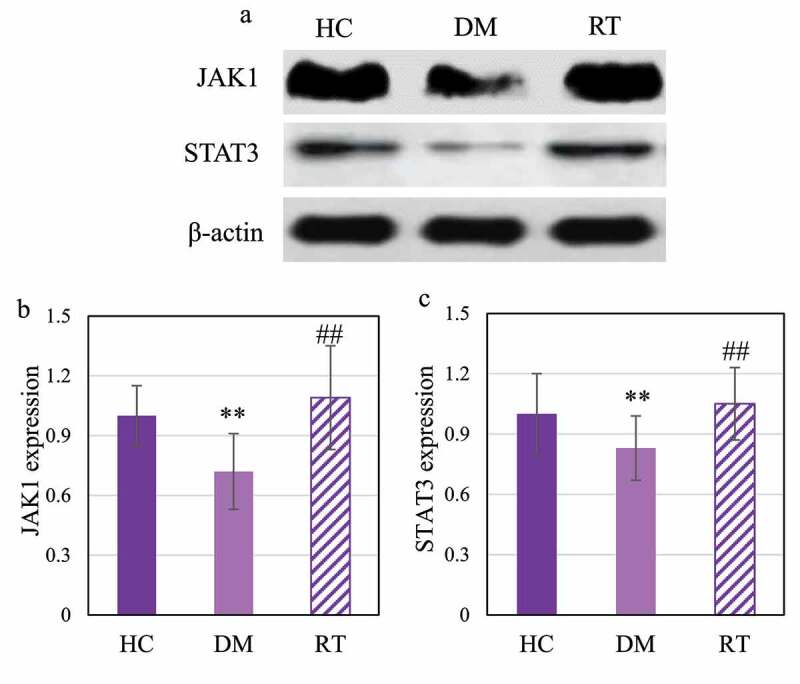
Comparison on JAK1 and STAT3 protein expression levels in the hippocampus of rats in each group.

Western blot was adopted to detect the effect of sodium valproate on the expression of JAK1 and STAT3 proteins in the hippocampus of rats with depression, and the results are shown in Figure 7. Figure 7(a) shows that JAK1 and STAT3 were dramatically reduced in the DM group. Analysis results on gray value of JAK1 and STAT3 protein expression are given in Figure 7(b,c). The DM group rats showed extremely reduced JAK1 and STAT3 protein expression levels in contrast to the HC group (*P* < 0.01) and the RT group (*P* < 0.05); while the levels in the HC group and the RT group were not significantly different (*P* > 0.05).

Figure A was the Western blot strip diagram; Figure B and Figure C showed the JAK1 protein expression level and the STAT3 protein expression level, respectively. ** and ## suggested *P* < 0.01 in contrast to HC and DM groups, respectively.

## 4. Discussion

Depression is a very common mental disorder with low mood and lack of interest as the main manifestation. It shows invisibility, and the course of the disease is long. In severe cases, it can lead to suicidal behavior of patients [17]. The pathogenesis of depression is very complex, and involves a variety of pathological changes. The current drugs used to treat depression are mainly focused on the function of monoamine transmitter system, but the effective rate of treatment is only about 60%–70%, and patients still have the risk of relapse after treatment [18]. Sodium valproate drugs are a class of antiepileptic and anticonvulsant drugs, which can improve the cognitive function and learning memory function disorders of animal models of Alzheimer’s disease by activating the Kv7 pathway [19]. This study was to explore the effects of sodium valproate on MRI features, neurological metabolism, and cognitive function disorders in the hippocampus of rats with depression caused by chronic stress.

Chronic stress can damage the cognitive function of the hippocampus and cause a variety of affective disorders [20]. The results of this study showed that the growth rate of body weight of tested rats was reduced obviously after chronic stress. Sugar water preference, forced swimming test, and tail suspension test are commonly used to evaluate depression-like behaviors in animal models [21]. The results of this study proved that the sugar water preference rate of rats after chronic restraint stress decreased tremendously, and the cumulative immobility time in the forced swimming test and tail suspension test increased. Such results suggest that chronic restraint stress can increase the depression-like behavior of rats, so the rats with depression model is successfully constructed. Morris water maze and NOR are common methods used to evaluate the cognitive function of hippocampus dependence [22]. After chronic restraint stress, the escape latency of rats was greatly prolonged, while the target quadrant stay time and number of crossings were extremely reduced, and the divergence index was decreased greatly in this study. After chronic restraint stress, MRI of rat hippocampus showed asymmetric and deepened brain split. It shows that chronic restraint stress stimulation can cause damage to dependent spatial learning and memory function in the hippocampus of rats, and reduce the ability of rats to distinguish new objects [23,24]. Such results are consistent with the conclusions of Wu et al. (2021) [25]. After modeling in this study, sodium valproate increased the sugar water preference rate of rats, shortened cumulative immobility time and escape latency, increased target quadrant stay time, number of crossings, and symmetrical MRI features of the hippocampus and normal split brain. Such results verified that Sodium valproate can improve the depression-like behavior of the rat model and its impairment of spatial learning and memory, and it can also increase the rat’s cognition of new objects.

MRS is a noninvasive MRI technology that can detect the biochemical changes of the living brain, and it can be used to explore changes in the brain structure and function of depression models [26]. The main peak signals in the 1 H-MRS spectrum are NAA, Glu, Cho, MI, and Cr. NAA/Cr can be undertaken as an evaluation index for neuronal dysfunction or neuron loss [27]. The results of this study showed that chronic restraint stress could reduce the levels of NAA/Cr, Glu/Cr, and Cho/Cr, while Sodium valproate could increase the levels of various indicators in the hippocampus of rats. These results suggest that chronic restraint stress causes obvious neuronal dysfunction in rats, and sodium valproate can improve the pathological changes of depression.

At present, many studies have confirmed that the levels of inflammatory factors such as IL-1β and IL-6 in the serum of patients with depression are dramatically increased [28,29]. It is similar to the results found in this study that the levels of IL-1β and IL-6 protein in the hippocampus of rats with depression increased. This suggests that the activation of inflammatory cytokines is involved in the occurrence of depression. JAK1/STAT3 is one of the most important cytokine signal transduction pathways, which is involved in the process of inflammation, cell damage, and cell apoptosis [30]. The results of this study indicated that the expression levels of JAK1 and STAT3 protein in the hippocampus of rats decreased after chronic restraint stress, while those increased after Sodium valproate treatment, and the protein levels of IL-1β and IL-6 decreased. Such results reveal that sodium valproate treatment can inhibit the activation of inflammatory factors and activate the activation of JAK1/STAT3 pathway to play the role of neuroprotection and functional restoration.

## 5. Conclusion

To explore the effects of antiepileptic drugs on MRI images, neurological cognition, and JAK1/STAT3 pathways in the hippocampus of rats with depression, this study established a rat model of depression and gave sodium valproate injection treatment on this basis. First, it explored the changes in depression-like behavior and cognitive dysfunction in rats. Then, the MRI images and MRS scans were adopted to analyze the changes in the structure of the rat’s hippocampus and the metabolism of neurobiochemical substances. Finally, the changes of inflammatory factors and JAK1/STAT3 pathway in the hippocampus of rats were detected. The results showed that sodium valproate can improve the depression-like and cognitive dysfunction of rats after chronic restraint stress by activating the JAK1/STAT3 pathway and inhibiting the activation of inflammatory factors. However, this study did not analyze whether different doses of sodium valproate would affect the treatment effect. Moreover, the sample size of this study was relatively small; and the physical condition of each rat was different, which was different. Therefore, whether Sodium valproate can activate the JAK1/STAT3 pathway each time required further research. It was expected to analyze the effects of different doses of sodium valproate on cognitive function disorders in rats with depression in the later stage.
